# Maximum Entropy Analysis of Flow Networks: Theoretical Foundation and Applications

**DOI:** 10.3390/e21080776

**Published:** 2019-08-08

**Authors:** Robert K. Niven, Markus Abel, Michael Schlegel, Steven H. Waldrip

**Affiliations:** 1 School of Engineering and Information Technology, The University of New South Wales, Northcott Drive, Canberra, ACT 2600, Australia; 2 Ambrosys GmbH, 14469 Potsdam, Germany; 3 Institute for Physics and Astrophysics, University of Potsdam, 14469 Potsdam, Germany; 4 Institut für Strömungsmechanik und Technische Akustik, Technische Universität Berlin, 10623 Berlin, Germany

**Keywords:** maximum entropy analysis, flow network, probabilistic inference

## Abstract

The concept of a “flow network”—a set of nodes and links which carries one or more flows—unites many different disciplines, including pipe flow, fluid flow, electrical, chemical reaction, ecological, epidemiological, neurological, communications, transportation, financial, economic and human social networks. This Feature Paper presents a generalized maximum entropy framework to infer the state of a flow network, including its flow rates and other properties, in probabilistic form. In this method, the network uncertainty is represented by a joint probability function over its unknowns, subject to all that is known. This gives a relative entropy function which is maximized, subject to the constraints, to determine the most probable or most representative state of the network. The constraints can include “observable” constraints on various parameters, “physical” constraints such as conservation laws and frictional properties, and “graphical” constraints arising from uncertainty in the network structure itself. Since the method is probabilistic, it enables the prediction of network properties when there is insufficient information to obtain a deterministic solution. The derived framework can incorporate nonlinear constraints or nonlinear interdependencies between variables, at the cost of requiring numerical solution. The theoretical foundations of the method are first presented, followed by its application to a variety of flow networks.

## 1. Introduction

The past decade witnessed a tremendous growth of interest in the structural and dynamic properties of networks, especially from the perspective of statistical physics, e.g., [1,2,3,4,5,6,7,8,9,10]. This was triggered by many new applications, e.g., the Internet and Web 2.0 applications, e.g., [11,12]; human social networks, e.g., [3,4,8]; electrical power networks with distributed generation and storage, e.g., [13,14]; land transport, air traffic and freight networks, e.g., [15,16,17,18,19]; complex dynamical systems and causal networks, e.g., [20,21,22]; nonequilibrium chemical reaction and biological networks, such as metabolic networks, e.g., [23,24,25]; ecological and global climate networks, e.g., [26,27]; and “networks of networks”, e.g., [28,29,30,31]. A literature review spanning all of these applications would be vast; we here defer to several reviews and monographs [3,4,5,7,9,19,21,30,31]. However, despite the breadth and depth of these studies, many fundamental questions on the analysis of networks have not been satisfactorily resolved. In particular, while many studies have analyzed the dynamics *of* networks, much less attention has been devoted to the analysis of dynamics *on* networks, or the combined problem of dynamics *on and by* networks. Despite several pioneering studies within several disciplines, a general probabilistic framework for the analysis of flows on networks subject to uncertainty has not been presented.

**Figure 1:**
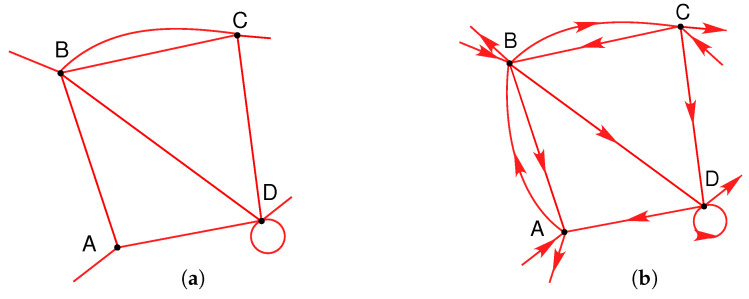
Graph representations of flow networks, with (**a**) undirected or (**b**) directed flows.

We here define a *flow network* as a set of nodes connected by links, which carries flow(s) of one or more quantities; typically but not exclusively of conserved quantities. Such networks can be represented by undirected or directed graph structures such as those illustrated in Figure 1a,b. Many flow networks will carry flows that are driven by or associated with differences in a physical field or potential, while in others, the demand is governed by parameters other than potentials. The concept of a flow network thus provides a unifying theoretical framework for the analysis of networks from many disparate disciplines, e.g., pipe flow, fluid flow, electrical, chemical reaction, ecological, epidemiological, neurological, communications, transportation, financial, economic and human social networks. Consider, for example, two types of flow network of importance to human society: (i) pipe flow networks used for the distribution of potable water and natural gas in populated areas, e.g., [32,33], and (ii) transportation networks used by motor vehicles, e.g., [15,16,17,18,19]. In the latter case, the network can have a physical manifestation such as a road or rail network, or could be a virtual (topological) network represented by aircraft or shipping routes. A third example of increasing interest is that of epidemiological networks, which govern the spread of disease, e.g., [34,35]; these commonly require interconnections between networks of different character, or “networks of networks” [28,29,30,31]. 

The aim of this study is to present a general framework for the analysis of any flow network based on the maximum entropy (MaxEnt) method [36,37,38,39,40,41], the fundamental tool of statistical physics. The method is founded on the generic (probabilistic or information-theoretic) definition of entropy, a measure of the spread of a probability distribution, which in thermodynamics has a deep connection to observable irreversible change. In the MaxEnt method, the user maximizes the entropy of a system, subject to any physical constraints, to infer its state, expressed in the form of a probability distribution. This can be post-processed to extract additional information on the system, such as its statistical moments. The power of the MaxEnt method lies in its ability to infer the state of an underconstrained (underdetermined) dynamical system, i.e., for which the number of unknowns exceeds the number of equations. In many situations it also enables a substantial reduction in model order. The classical example of this utility is demonstrated by chemical thermodynamics, for which the MaxEnt method generally furnishes a 23-order-of-magnitude reduction compared to the underlying (molecular or canonical) dynamical system. In addition to its outstanding successes in statistical thermodynamics, over the past few decades, the MaxEnt method provided new insights in many other fields of research, throughout all branches of science, engineering, mathematics and the social sciences [38,41,42,43].

We first sketch the relationship between the present framework and conventional thermodynamics: whereas in many problems of physics, a system will consist of a discrete or continuous set of (macro)states embedded in a continuous (physical or phase) space, in a flow network the geometry is partly discretized. Adopting a statistical physics formulation, we assign state variables to the flow rates through each internal and external link of the network, and also (if defined) to the potentials at each node. A network ensemble can then be conceptualized by considering all copies of the system at different times or with different initial conditions, as is usual in statistical physics. The (generic) entropy of the system can then be defined using the Boltzmann equation H=lnP [44,45,46], where P is the governing probability distribution, which in this case expresses the allocation of entities (flow rate and potential state variables) to states (their possible values). For discrete, distinguishable entities and states, a multinomial distribution results: $P=N!\prod_{i}q_{i}^{n_{i}}/n_{i}!$, where $n_{i}$ entities are allocated to the *i*th state, $q_{i}$ is the prior probability of allocating an entity to the *i*th state, and N is the total number of entities. Here we note that the prior probability $q_{i}$ represents the *i*th state of the “unconstrained” system, and is needed to account for states of unequal weighting (degeneracy). In the asymptotic limits N→∞ and $n_{i}/N\to p_{i}$, this gives the relative entropy $H=-\sum_{i}p_{i}\ln\left(p_{i}/q_{i}\right)$ [38,47,48]. The network will also be subject to a variety of constraints, in the form of specified values of various statistical moments, physical laws and network properties; these must be incorporated into the optimization procedure. Maximizing the relative entropy H, subject to the constraints, is then equivalent to maximizing P (in the asymptotic limit) subject to the same constraints, to give the most probable state of the network. From the information-theoretic perspective, this can also be interpreted as the least informative description of the network, or that which contains the least information subject to the constraints [36,37,38,41]. In the present study, for mathematical convenience we consider continuous flows on a discretized network, giving a continuous, multivariate form of the above apparatus.

This work is set out as follows. In Section 2, we define the generalized flow network under consideration, including a standard set of specifications applicable to potential-flow and transportation networks. In Section 3, we provide a general formulation for the probability distribution, entropy and constraints for this standard network, and work through the operation of the MaxEnt method to its main findings. For this we examine the handling of nonlinear moment constraints and interdependencies, not usually examined under the MaxEnt framework. We also discuss the role of time in the MaxEnt formulation. In Section 4, we discuss the application of the method to a variety of flow networks. A brief comparison between the MaxEnt method and Bayesian inference is provided in Section 5. The conclusions are then summarized in Section 6.

## 2. Network Specifications

Consider a generalized flow network such as one of those represented in Figure 1a,b. For maximum generality, we consider all possible undirected graphs (with unspecified flow directions), directed graphs or digraphs (with fixed flow directions), simple graphs (no self-loops or multiple connections), loop-graphs (with self-loops) and multigraphs (with multiple connections). The multigraphs can be further classified into undirected and directed forms, and those without or with loops. Weighted graphs are treated as continuous forms of multigraphs. We do not specifically discuss bipartite graphs or networks of networks, but these can be analyzed by extension of the formulation given here.

We first consider a flow network defined by the following set of quantitative specifications, here termed the “standard set”:

(1) A set of $N\in N\cup 0=N_{0}$ vertices (nodes), with index *i* or *j*.
(2) A set of $M\in N_{0}$ internal edges (including loop edges) between pairs of nodes, commonly represented by an N×N adjacency matrix A, in which each element $A_{ij}$ indicates the connectivity between nodes *i* and *j*. On an undirected graph, $A_{ij}\in \left\{0,1\right\}$, indicating Boolean connectivity (hence A is symmetric with a zero diagonal), so $\sum_{i=1}^{N}\sum_{j=1}^{N}A_{ij}=\left[2M,2M-L\right]$ respectively for undirected simple graphs and undirected loop-graphs, where $L\in N_{0}$ is the number of self-loops. On a digraph, $A_{ij}\in \left\{0,1\right\}$ refers strictly to the connection from node *i* to *j*, thus incorporating the flow direction (so A can be asymmetric), hence $\sum_{i=1}^{N}\sum_{j=1}^{N}A_{ij}=M$. On a multigraph, $A_{ij}\in N_{0}$ indicates the number of edges from node *i* to *j*, whence $\sum_{i=1}^{N}\sum_{j=1}^{N}A_{ij}=\left[2M,2M-L,M\right]$ respectively for undirected multigraphs, undirected loop multigraphs and multidigraphs. For weighted graphs, $A_{ij}\in \left[0,\infty \right)=R_{0}^{+}$ (or more generally $A_{ij}\in R$), representing the weight of each edge. Double summation then gives $\sum_{i=1}^{N}\sum_{j=1}^{N}A_{ij}=\left[2W,2W-W_{L},W\right]$ respectively for undirected, undirected loop and directed weighted graphs, where $W,W_{L}\in R_{0}^{+}$ (or more generally $W,W_{L}\in R$) are respectively the total weight and the weight of self-loops.
(3) A set of $P\in N_{0}$ external edges (links) to nodes, represented by an *N*-dimensional external adjacency vector U, in which each element $U_{i}\in N_{0}$ indicates the number of external links to node *i*. For undirected graphs, digraphs and multigraphs, $P=\sum_{i=1}^{N}U_{i}$. (While not implemented here, the external links could alternatively be represented by *P* connections to a fictitious external node, allowing all links to be united into an (N+1)×(N+1) augmented adjacency matrix $\tilde{A}$ [24,33].) For a weighted graph, if the external edges are also weighted then $W_{P}=\sum_{i=1}^{N}U_{i}$, where $W_{P}\in R_{0}^{+}$ (or more generally $W_{P}\in R$) is the total weight of external edges.
(4) A set of *M* internal flow rates $Q_{ij}$, i,j∈{1,…,N}, of some countable quantity B from nodes *i* to *j* along the ijth edge, measured in units of B s${}^{-1}$. In general, the flow rates will be functions of time and/or the graph ensemble. For a simple graph, they can be grouped into the N×N flow rate matrix Q, with nonexistent edges handled by assigning $Q_{ij}=0$ or $Q_{ij}=\mathrm{undefined}$. (Alternatively, the admissible flow rates $Q_{n}$ can be stacked into a flow rate vector Q, for which the ijth and *n*th edges are connected by a lookup table [32,33].) In addition:
  (a) On an undirected graph, the non-diagonal flow rates $Q_{ij}\in R,\forall i\neq j$, are antisymmetric $Q_{ij}=-Q_{ji}$ and can reverse direction. In contrast, on a digraph they are strictly nonnegative $Q_{ij}\in R_{0}^{+}$ and need not be correlated $Q_{ij}⪋Q_{ji}$. We recognize that in all networks, the flows are quantized (especially significant for transport networks), but for maximum generality, we here adopt the continuum assumption with real-valued flow rates.
  (b) A graph with self-loops can have $Q_{ii}\in R_{0}^{+}$, i.e., $Q_{ii}\geq 0$. Such flows are incompatible with potential flow networks, but can be realized in other systems such as transport networks.
  (c) On a multigraph, there may be up to $K=\max\left(A_{ij}\right)\in N_{0}$ connections from node *i* to *j*, with flow rates labelled $Q_{ij(k)}$ for k∈{1,…,K}. These can be united into the N×N×K third-order matrix Q, assigning $Q_{ij(k)}=0$ or $Q_{ij(k)}=\mathrm{undefined}$ for nonexistent edges. If summed along the *k*th direction, this gives an N×N total internal flow rate matrix $\hat{Q}$, with entries $Q_{ij}=\sum_{k=1}^{K}Q_{ij(k)}$.
  (d) For networks with alternating electrical currents, it is convenient to consider complex flow rates (phasors) $Q_{ij(k)}\in C$, commonly expanded as $Q_{ij(k)}=I_{ij(k)}=\left|I_{ij(k)}\right|\exp\left(ı\psi_{ij(k)}\right)$, where $|I_{ij(k)}|$ is the current amplitude, $\psi_{ij(k)}$ is the phase and $ı=\sqrt{-1}$.
  (e) For multidimensional flows, we define $Q_{ij(k)c}$ as the flow of species c∈{1,…,C} on the ij(k)th edge, where *C* is the total number of species (e.g., cars, trucks and buses on a road network, or independent chemical species on a chemical reaction network). These can be collated into the N×N×K×C fourth-order matrix Q, which if summed along the *k*th direction gives the N×N×C total internal flow rate matrix $\hat{Q}$. For brevity, we refer to flows of a *C*-dimensional vector quantity B with components $B_{c}\in \left\{B_{1},\ldots ,B_{C}\right\}$.
  (f) In some flow networks, a given edge into a node may be connected only to some outward edges (e.g., certain traffic junctions in cities, or protected nodes in electrical networks). Such nodes must be treated as graphical objects in their own right, leading to embedded networks-of-networks. Such complications, while important, are not examined further here.
(5) A set of *P* external flow rates $\Theta_{i(m)}\in R$, i∈{1,…,N}, m∈{1,…,O}, where $O=\max(U_{i})$, denoting flow on the *m*th external link to node *i*, here defined positive if an inward flow. These flow rates are also measured in units of B s${}^{-1}$, and can be grouped into the N×O matrix Θ, again assigning $\Theta_{i(m)}=0$ or $\Theta_{i(m)}=\mathrm{undefined}$ for nonexistent external links. Furthermore:
  (a) For networks with a maximum of one external link to each node (O=1), we need only consider the *N*-vector Θ of external flow rates $\Theta_{i}$.
  (b) For alternating current networks, we consider complex external flow rates (phasors) $\Theta_{i(m)}=\left|\Theta_{i(m)}\right|\exp\left(ı\eta_{i(m)}\right)\in C$, where $|\Theta_{i(m)}|$ is the external current amplitude and $\eta_{i(m)}$ the phase.
  (c) For multidimensional flows, we consider the flow rate $\Theta_{i(m)c}$ of each species *c*, which can be assembled into the N×O×C external flow rate matrix Θ. This can be summed along the *m* direction to give the N×C total external flow rate matrix $\hat{\Theta }$, with entries $\sum_{m=1}^{O}\Theta_{i(m)c}$.
(6) For potential flow systems: a set of *N* potentials $E_{i}\in R$ at each node i∈{1,…,N}, united into the *N*-dimensional vector E. For alternating current networks, we consider complex potentials (phasors) $E_{i}\in C$, commonly written as $E_{i}=V_{i}=\left|V_{i}\right|\exp\left(ı\phi_{i}\right)$, where $|V_{i}|$ is the electrical potential amplitude and $\phi_{i}$ is the phase. For multidimensional flows, we consider the independent potentials $E_{ic}$, assembled into the N×C matrix E.
(7) For potential flow systems: a set of *M* potential losses (negative differences), on a simple graph given by:
  (1) $$▴E_{ij}=-\Delta E_{ij}=E_{i}-E_{j},\;\forall i,j$$
  These are generally interpreted as driving forces for the flow rates $Q_{ij}$, measured in units of a difference or gradient in the intensive variable conjugate to B. Examples include losses in pressure, electrical potential, temperature (or reciprocal temperature) and chemical potential (or chemical potential divided by temperature). For simple graphs, these can be assembled into the N×N matrix ▴E. In a multigraph, the potential losses are independent of subindex *k*:
  (2) $$▴E_{ij(k)}=▴E_{ij},\;\forall i,j,k$$
  but it is useful computationally to retain all terms in an N×N×K matrix ▴E. For multicomponent flows, the potential losses $▴E_{ij(k)c}=-\Delta E_{ij(k)c}=E_{ic}-E_{jc}$ give the N×N×K×C matrix ▴E.
(8) For potential flow systems: a set of *M* resistance or constitutive relations for each edge, which for purely local dependencies can be written as:
  (3) $$▴E_{ij(k)}=-\Delta E_{ij(k)}=R_{ij(k)}\left(Q_{ij(k)}\right),\;\forall i,j,k$$
  where $R_{ij(k)}$ is the ij(k)th resistance relation. For example, in electrical circuits, (3) give linear relations $▴E_{ij(k)}=R_{ij(k)}Q_{ij(k)}$ (Ohm’s law), where $R_{ij(k)}$ is the resistance (for direct currents) or impedance (for alternating currents) of the ij(k)th edge. In pipe flow networks, (3) are often written as power law relations $▴E_{ij(k)}=X_{ij(k)}Q_{ij(k)}\;\left|\;Q_{ij(k)}\;\right|{\;}^{\alpha -1}$ (Blasius’ law), where $X_{ij(k)}$ is a parameter and 1≤α≤2 is a coefficient [49,50], or can be expressed in more complicated forms such as the Colebrook equation [51]. Some resistance relations may not be functions: i.e., they may allow multiple solutions. For multidimensional flows, (3) applied to the *c*th pair of flow rates and potential losses gives:
  (4) $$▴E_{ij(k)c}=R_{ij(k)c}\left(Q_{ij(k)c}\right),\;\forall i,j,k,c$$
  In multidimensional flows, there is also the possibility of cross-phenomenological transport processes, e.g., of thermodiffusive, thermoelectric, electrokinetic, electroosmotic or galvanomagnetic phenomena, e.g., [52,53,54,55,56]. These are usually represented by linear Onsager relations, valid close to equilibrium, of the form:
  (5) $$▴E_{ij(k)b}=\sum_{c=1}^{C}R_{ij(k)bc}Q_{ij(k)c},\;\forall i,j,k,b$$
  in which $R_{ij(k)bc}$ is the bcth phenomenological resistance, which will satisfy reciprocity $R_{ij(k)bc}=R_{ij(k)cb}$. However, in general these will require the (nonlinear) relations:
  (6) $$▴E_{ij(k)b}=\sum_{c=1}^{C}R_{ij(k)bc}\left(Q_{ij(k)c}\right),\;\forall i,j,k,b$$
  in which $R_{ij(k)bc}$ is now the bcth phenomenological resistance function. The connections between reciprocal functions may be complicated. Equations (3)–(6) can be assembled into the function:
  (7) ▴E=R(Q)
  where R is an N×N×K×C resistance operator, which in general may contain non-local effects, cross-phenomenological effects and other dependencies.

Combining the above points, we consider a flow network defined by the standard set of specifications:

(8) S={N,M,P,K,O,A,U,C,B,Q,Θ,E,▴E,R}

which from (1)–(7) and various other constraints—to be discussed—will involve some functional interdependencies. It is convenient here to amalgamate all graph properties into the discrete function:

(9) G=G(N,M,P,K,O,A,U)

and also to remove the redundancy between potentials and potential differences, to give the set:

(10) $$S=\{G,C,B,Q,\Theta ,▴E,E^{⦵},R\}$$

where $E^{⦵}$ is a vector of reference potentials $E_{c}^{⦵}$ at a nominated node.

We note that many networks—especially in transportation—do not have potentials, potential differences or resistances. Such networks can be examined using the above standard set (10), with the demand handled by the use of cost or travel time functions instead of potentials, in many cases augmented by various strategies or optimization schemes for route selection [15,16,18].

## 3. Maximum Entropy Analysis

### 3.1. Overview

We now consider the analysis of flow networks by the maximum entropy (MaxEnt) method. This always consists of the following steps [38]:

(1) Definition of a joint probability and/or probability density function (pdf), to quantify all uncertainties in the specification of a given system;
(2) Definition of a relative entropy function for the network, based on this probabilistic representation;
(3) Incorporation of information about the *a priori* distribution of the system over its parameter space, in the form of a prior probability and/or pdf;
(4) Encoding of other background information, for example any physical laws or known parameter values, in the form of constraints;
(5) Maximization of the relative entropy function, subject to the prior and constraints, to predict the state of the system.

Philosophically, the MaxEnt algorithm has been shown to select the most probable description of the system [44,45,46], or its least informative description [36,37,38,41], consistent with the constraints. This is generally interpreted as the stationary state of the system, equivalent to its “typical” or “most representative” state. In typical thermodynamic systems involving isolated systems or canonical ensembles—for which the probabilities and constraints are defined over the contents of the system or the ensemble—the stationary state is interpreted as the equilibrium position of the system, e.g., [57,58,59,60]. However, in forced flow systems such as flow networks—in which the probabilities and constraints are defined over constant flow rates or fluxes into or through part of the system—the stationary state must instead be interpreted as the steady state of the system [61,62,63]. (We note that the term “steady-state” is somewhat misleading, since it refers only to the mean flow and not its fluctuations; a steady-state flow need not be steady in time, only in the mean.) More elaborate formulations involving time-dependent constraints are discussed further in Section 3.6.

Each step of the MaxEnt framework, applied to the analysis of flow networks, is discussed in turn.

### 3.2. Uncertainty and Probabilistic Representation

A flow network is always subject to some uncertainty associated with a lack of knowledge of its specification. For example, there may be uncertainty in the known values of its instantaneous flow rates Q and potential differences ΔE; in its controlling external flows Θ; in its more fundamental properties such as the resistance functions R; or even concerning the existence of specific internal or external links (represented by A or U) or the number of nodes *N*. The maximum entropy (MaxEnt) method of Jaynes provides a rigorous technique with which to predict the state of the network, despite these inherent uncertainties in its specification.

We must first represent the uncertainties in quantitative form. Following the well-established line of reasoning developed by Bayes [64], Laplace [65] and many others, extended by Polya [66,67], Cox [68] and Jaynes [38], the uncertainty (or degree of belief) in a discrete parameter should be expressed as a probability. Writing $\Upsilon_{n}$ for a discrete random variable which takes values *n* over the domain $\Omega_{n}\subseteq N$ or $\Omega_{n}\subseteq Z$, this probability is defined by:

(11) $$p\left(n\;|\;I\right)=\mathrm{Prob}.\left(\Upsilon_{n}=n\;|\;I\right)$$

Some authors refer to this as a probability mass function (pmf) [69]. Equation (11) is conditioned on the background information *I*, which contains all that is known from the problem specification (including the parameter space, prior and constraints). Equation (11) will satisfy normalization, $\sum_{n\in \Omega_{n}}p\left(n\;|\;I\right)=1$.

Alternatively, for a real parameter *x*, the uncertainty should be expressed as a pdf. For a continuous random variable $\Upsilon_{x}$ which takes values *x* over the domain $\Omega_{x}\subseteq R$, this is defined by the probability:

(12) $$p\left(x\;|\;I\right)dx=\mathrm{Prob}.\left(x\leq \Upsilon_{x}\leq x+dx\;|\;I\right)$$

again conditioned on some background information *I*. This will again satisfy normalization $\int_{\Omega_{x}}p\left(x\;|\;I\right)dx=1$. Extending this to an *s*-dimensional continuous vector or matrix quantity x, we can write the joint probability:

(13) $$\begin{array}{c} p\left(x\;|\;I\right)dx=\mathrm{Prob}.\left(x\leq \Upsilon_{x}\leq x+dx\;\;|\;\;I\right)=\mathrm{Prob}.\left(\begin{array}{c} x_{1}\leq \Upsilon_{x_{1}}\leq x_{1}+dx_{1}\\ \vdots \\ x_{s}\leq \Upsilon_{x_{s}}\leq x_{s}+dx_{s} \end{array}|\;I\right) \end{array}$$

in which $dx=\prod_{i=1}^{s}dx_{i}$ is the product of differentials of all terms. We can also consider a joint probability containing both discrete and continuous variables, thus invoking a mixed probability mass and density function, for simplicity also referred to here as a pdf.

Accordingly, the uncertainty in a flow network defined by the standard set of specifications (10) is given by the joint probability:

(14) $$p\left(S\;|\;I\right)=p\left(G,C,B,Q,\Theta ,▴E,E^{⦵},R\;|\;I\right)dQ\;d\Theta \;d▴E\;dE^{⦵}\;dR$$

This definition accounts for the fact that G, *C* and B are discrete, while the other variables are continuous. Equation (14) is again conditioned on the background information *I*. In (14), the configurational specifications are listed first, but this does not imply any priority order. If some parameters are not required, e.g., if there is no need to consider potentials or potential differences (such as in a transport network), these can be omitted from (14).

In consequence, for any given problem involving a flow network, we can write a joint pdf to describe its uncertainty over the set of unknown parameters, conditional on those which are known. For example, if the network configuration, flow species, external flow rates, reference potentials and resistance functions are known, but there is uncertainty over the internal flow rates and potential differences, we adopt the joint conditional pdf:

(15) $$p(Q,▴E|\;G,C,B,\Theta ,E^{⦵},R,I)=\frac{p(G,C,B,Q,\Theta ,▴E,E^{⦵},R\;|\;I)}{p(G,C,B,\Theta ,E^{⦵},R\;|\;I)}$$

which as indicated, is equivalent to the original pdf (14) divided by the pdf of known parameters. The latter is defined by:

(16) $$\begin{array}{cc} p(G,C,B,\Theta ,E^{⦵},R\;|\;I) & =\int_{\Omega_{Q}}\;\int_{\Omega_{▴E}}\;p\left(G,C,B,Q,\Theta ,▴E,E^{⦵},R\;|\;I\right)\;dQ\;d▴E \end{array}$$

i.e., by marginalization (summation or integration) of the original pdf (14) over the unknown parameters. For simplicity, in the following we adopt the notation:

(17) $$p\left(f\;|\;g,I\right)=\frac{p(f,g|I)}{p(g|I)}=\frac{p(f,g|I)}{\underset{\Omega_{f}}{⨋}p\left(f,g\;|\;I\right)}$$

where f and g respectively denote the sets of unknown and known parameters and operators, while ${⨋}_{\Omega_{f}}$ indicates summation or integration, as appropriate, with respect to the discrete or continuous parameters within $\Omega_{f}$, the differentials being implicit [69].

Based on the above analysis, we can therefore speak of the *level of description* of a flow network, given by the sets of unknown and known parameters and operators in its specifying pdf. At the lowest level of description, only the background information *I* is known, giving the pdf (14). At the next few levels, successively more information will be included, for example on its graphical properties G. At higher levels, knowledge of other features, such as the resistance functions R, will also be included. The flow network specified by (15) is posed at an even higher level of description. At the highest level of description, a flow network can be said to be *fully specified*; i.e., its description—including the prior and constraints embedded within *I*—furnishes sufficient information to fully determine all properties, including all flow rates within each edge and (if included) all potentials at each node. At this level, there is no need to invoke a probabilistic formulation, since the problem is now deterministic.

For some networks, the conditional pdf (17) might be separable into distinct sums and/or integrals over particular parameters or subsets, allowing dramatic simplification. However, the above probabilistic representation will always remain valid, regardless of whether such separability is possible.

### 3.3. Entropy and Prior Probabilities

The *relative entropy* of a flow network is defined over all uncertainties in the network, and therefore follows directly from its probabilistic representation (17). From the Boltzmann approach discussed in the Introduction, we adopt the multivariate relative entropy (negative Kullback–Leibler divergence [47]), in shorthand form:

(18) $$H=-\underset{\Omega_{f}}{⨋}p\left(f\;|\;g,I\right)\ln\frac{p(f\;|\;g,I)}{q(f\;|\;g,I)}$$

where q(f|g,I) is the *joint prior pdf* or simply the *prior*, defined at the same level of description as the problem specification (17). The sums and/or integrals in (18) are evaluated over the domain $\Omega_{f}$ of all unknown parameters.

The prior represents one part of the background information *I*, providing a probabilistic baseline or “reference distribution” for the flow network in the absence of constraints. It thereby accounts for different *a priori* weights of the parameter values. Indeed, for any continuous parameters, to ensure dimensional consistency and scale invariance, the prior cannot be omitted [37,38].

In many MaxEnt analyses, the prior is often assumed constant by default, equivalent to adopting the *improper prior* q=1 [38]. If; however, one knows that a random variable is not distributed uniformly over its parameter space—if it is more probable, *a priori*, for certain values or subdomains to be occupied—then this information must be included in the prior. In thermodynamics, this is referred to as the *degeneracy*. Similar (opposite) considerations apply to less probable values or domains, or those which must be strictly excluded. Sometimes these restrictions can be formulated as strict constraints rather than imposed within the prior. An illustrative example is the adjacency matrix A of an *N*-node multigraph, which is drawn from the domain $\Omega_{A}={N_{0}}^{N\times N}$. In the absence of a prior or some other restriction, the MaxEnt algorithm will assign equal weightings to all graphs within this ensemble, i.e., will be heavily biased towards graphs with infinite numbers of edges. Another example is a constructed potential flow network such as an electrical or pipe flow network: *a priori*, no edge can carry an infinite flow rate, in either direction. Unless this information is encoded within a prior, the MaxEnt algorithm will be biased towards the consideration of extremely high (unphysical) flow rates. It is, therefore, crucial to incorporate any restrictions on the parameter specifications within the problem formulation, to avoid inferring a solution for a system which is far from the network of interest.

### 3.4. Moment Constraints

The second component of the background information *I* consists of constraints upon or between parameters, in MaxEnt analysis usually expressed in the form of mathematical moments (expectations). These always include normalization of the joint pdf:

(19) $$\langle 1\rangle =\underset{\Omega_{f}}{⨋}p\left(f\;|\;g,I\right)=1$$

If the problem is formulated using several probabilities or pdfs, each must be normalized. A flow network may also be subject to *linear global constraints* on various functions r(f) of the unknown parameters—which could be scalars, vectors or in matrix form—summed and/or integrated with respect to all unknowns:

(20) $$\langle r\rangle =\underset{\Omega_{f}}{⨋}r\left(f\right)\;p\left(f\;|\;g,I\right)=v_{r}$$

where $v_{r}$ indicates some specified value of *r*. Some flow networks may also have *intermediate* or *local constraints*, in which the expectation is calculated over a lower-dimensional projection h=π(f) of the unknowns:

(21) $$⟦r{⟧}_{h}=\underset{\Omega_{h}\;\subset \;\Omega_{f}}{⨋}r\left(h\right)\;p\left(f\;|\;g,I\right)=v_{r}$$

These are less mathematically tractable, being functions of the remaining unknowns f\h. Many flow networks may also be subject to *nonlinear global constraints* of the form:

(22) $$F\left(\langle r\rangle \right)=F\left[\underset{\Omega_{f}}{⨋}r\left(f\right)\;p\left(f\;|\;g,I\right)\right]=0$$

where *F* is a nonlinear function of the expectations of r(f), whence in general F(〈r〉)≠〈F(r)〉. It is also possible to have *nonlinear local constraints*, defined by

(23) $$F\left(⟦r{⟧}_{h}\right)=F\left[\underset{\Omega_{h}\;\subset \;\Omega_{f}}{⨋}r(h)\;p\left(f\;|\;g,I\right)\right]=0$$

Finally, any of the above constraints could be imposed as an inequality, for example for the linear global constraints:

(24) $$\langle r\rangle =\underset{\Omega_{f}}{⨋}r\left(f\right)\;p\left(f\;|\;g,I\right)\leq v_{r}\;\mathrm{or}\;\langle r\rangle =\underset{\Omega_{f}}{⨋}r\left(f\right)\;p\left(f\;|\;g,I\right)\geq v_{r}$$

In this study, both linear and nonlinear global equality constraints are examined in detail, and we also comment on inequality constraints. In principle, many other kinds of constraints can be included in the MaxEnt method, although moment constraints are best suited to extremization by the method of the calculus of variations.

Physically, the moment constraints (20)–(21) are usually identified with the time average:

(25) $$\bar{r}=\underset{\Omega_{t}}{⨋}r\left(t\right)p\left(t|I\right)$$

where *t* is the discrete or continuous time, p(t|I) is the probability or pdf of time and $\Omega_{t}$ is the time domain, or alternatively the ensemble average:

(26) $$\tilde{r}=\underset{\Omega_{\rho }}{⨋}r\left(\rho \right)p\left(\rho |I\right)$$

where ρ is the discrete index or continuous density of individual realizations, p(ρ|I) is the probability or pdf of a realization and $\Omega_{\rho }$ is the domain of realizations. These are not the only viable choices; indeed, moment constraints can be identified with any function which satisfies the properties of a Reynolds average [70].

We therefore consider three categories of moment constraints on a flow network: (i) those arising from specified moments of various physical parameters, e.g., means, variances or higher-order moments of particular flow rates or potential differences, as defined by (20)–(21); (ii) those imposed by physical laws, in particular the conservation laws (Kirchhoff’s laws) and resistance functions; and (iii) those arising from constraints on the network properties (the graph ensemble). Philosophically, the physical laws and graphical constraints are appropriately imposed in the mean, to allow for fluctuations about their mean values; such analysis is particularly well suited to the MaxEnt framework.

Consider the latter two categories of constraints, for a flow network defined by the standard set of specifications (10). Firstly, Kirchhoff’s laws can be written as:

(1) *Kirchhoff’s first law*: Applied in the mean, this states that the mean flow rate of each species *c* through each node *i* will be zero at steady state. For an undirected graph, this can be written as the sums of inflows and outflows:
  (27) $$\begin{array}{cc} 0 & =\sum_{m=1}^{O}\langle \Theta_{i(m)c}\rangle +\frac{1}{2}\sum_{j=1}^{N}\sum_{k=1}^{K}\left(\langle Q_{ji(k)c}\rangle -\langle Q_{ij(k)c}\rangle \right),\;\forall i,c\\ & =\sum_{m=1}^{O}\langle \Theta_{i(m)c}\rangle +\sum_{k=1}^{K}\langle Q_{ii(k)c}\rangle -\sum_{j=1}^{N}\sum_{k=1}^{K}\langle Q_{ij(k)c}\rangle ,\;\forall i,c \end{array}$$
  using $\langle Q_{ji(k)c}\rangle =-\langle Q_{ij(k)c}\rangle$ except for i=j. On a directed graph, the flow paths into and out of each node must be counted separately:
  (28) $$0=\sum_{m=1}^{O}\langle \Theta_{i(m)c}\rangle +\sum_{j=1}^{N}\sum_{k=1}^{K}\left(\langle Q_{ji(k)c}\rangle -\langle Q_{ij(k)c}\rangle \right),\;\forall i,c$$
  Reversing the sum and expectation operators, (27)–(28) can be assembled into the N×C matrix equation:
  (29) $$\langle \hat{\Theta }\rangle +\epsilon \;\left({\langle \hat{Q}\rangle }^{⊤}-\langle \hat{Q}\rangle \right)\;1=\langle \hat{\Theta }\rangle +\epsilon \;\Phi \left(\langle \hat{Q}\rangle \right)=0$$
  where 0 and 1 are vectors or matrices of 0s or 1s of appropriate dimension, *⊤* denotes the transpose, $\epsilon =\frac{1}{2}$ for an undirected graph and 1 for a directed graph, and Φ is an N×C operator on $\langle \hat{Q}\rangle$. Equation (29) makes use of the *k*-summed flow rate matrices $\hat{\Theta }$ and $\hat{Q}$, with the latter viewed from its N×N face, so the bracketed term accounts for the row and column sums of $\hat{Q}$ respectively.
(2) *Kirchhoff’s second law:* Applied in the mean, this states that at steady state, the mean potential losses must be in balance around each connected loop, or equivalently that the mean potentials at each node must be constant. For a flow network with multidimensional potentials, the first definition gives an expression for each loop (cycle) *ℓ* on the network:
  (30) $$\sum_{ij(k)\in \ell }\langle ▴E_{ij(k)c}\rangle =0,\;\forall \ell$$
  in which the potential losses are added in the assigned direction of *ℓ*. In a multigraph, (30) applies to each parallel edge k∈{1,…,K}, while in a directed graph, the two loop orientations (clockwise or anticlockwise) can be independent. A search algorithm is required to identify a maximal set of Λ independent loops, expressed by the N×N×K loop adjacency matrices $M_{\ell }$ for ℓ∈{1,…,Λ}, containing elements $M_{ij(k)\ell }\in \left\{-1,0,1\right\}$ to indicate the presence and orientation of each edge in the loop. These can be stacked into the N×N×K×Λ loop adjacency matrix M. Equation (30) can then be rewritten as the Λ×C matrix equation:
  (31) $$M⊘\langle ▴E\rangle =\left[\sum_{\mathrm{all}\;ij(k)}M_{ij(k)\ell }\;\langle ▴E_{ij(k)c}\rangle \right]=0$$
  where ⊘ is a contraction product over the common indices of two matrices, given by the sum of their element-wise products, which subsumes the vector scalar product (dot product) for vectors and the tensor scalar product (double dot product) for second order tensors. In some networks, additional terms (e.g., reservoir potentials, pump heads, electrical source potentials, etc.) may also appear in (30)–(31).

Secondly, the resistance function constraints (7) can be imposed either (i) strictly, thereby eliminating the potential differences as independent variables; or (ii) in the mean, based on the nonlinear relation:

(32) 〈▴E〉=R(〈Q〉)

In this case, ▴E must be included as an unknown within f. We note that the form of (32), rather than 〈▴E〉=〈R(Q)〉, is necessary to ensure consistency between the MaxEnt and deterministic solutions for a fully determined problem, when calculated using mean potential differences and flow rates. For alternating currents in electrical systems, (32) should instead be formulated in terms of root-mean-square quantities, thus connecting the second moments, $\langle {\left(▴E\right)}^{2}\rangle =R\left(\langle Q^{2}\rangle \right)$). Thirdly, in some networks there may be uncertainty in the number *C* or identities B of the flow species, necessitating their inclusion in f and the imposition of species constraints, for example:

(33) Ψ(〈C〉)=0

where Ψ is a vector or matrix operator. Finally, the graphical constraints could take many forms, depending on the problem specification. These again divide into (i) strict constraints, for example on the number of nodes or their connections, and (ii) weaker constraints such as moment constraints, for example on the expected degrees of certain nodes. The strict graphical constraints can be written as:

(34) Γ(G)=0

where Γ is a vector or matrix operator and G represents the properties of an individual graph. These relations can be included directly in the problem formulation *I* and do not require extremization. On the other hand, graphical moment constraints can be written in moment form as:

(35) Ξ(〈G〉)=0

where Ξ is another vector or matrix operator, based on the expected properties 〈G〉 of the graph ensemble. We see that (34) defines a “microcanonical graph ensemble”, whereas (35) can be interpreted as a “canonical graph ensemble”. In either case (34)–(35), the loop adjacency matrix M will be a function of the graph properties, respectively G or 〈G〉. In more complicated networks, there may also be coupling constraints between the graphical properties and physical parameters.

### 3.5. Extended MaxEnt Algorithm

We now provide an extended formulation of Jaynes’ MaxEnt algorithm, based on the relative entropy (18) subject to normalization (19), linear (20) and nonlinear (22) global constraints. Collecting the latter into generic stacked vector or matrix forms $\langle r\rangle =v_{r}$ and F(〈r〉)=0 respectively, we write the Lagrangian [36,37,38,41]:

(36) $$\begin{array}{cc} L & =-\underset{\Omega_{f}}{⨋}p\left(f\;|\;g,I\right)\ln\frac{p(f\;|\;g,I)}{q(f\;|\;g,I)}-\kappa \left(\underset{\Omega_{f}}{⨋}p\left(f\;|\;g,I\right)-1\right)-\eta ⊘\left(\underset{\Omega_{f}}{⨋}r\left(f\right)\;p\left(f\;|\;g,I\right)-v_{r}\right)-\zeta ⊘F\left(\langle r\rangle \right) \end{array}$$

where κ, η and ζ are Lagrangian multipliers respectively for each type of constraint, of compatible order and dimension to enable contraction into scalars. Applying the calculus of variations, we set the total variation of (36) to zero:

(37) $$\begin{array}{c} \delta L=0=\frac{\partial L}{\partial p(f\;|\;g,I)}\delta p\left(f\;|\;g,I\right) \end{array}$$

Since each δp can be non-zero, this gives ∂L/∂p=0 for each $f\in \Omega_{f}$. Taking the functional derivative of (36) with respect to *p*, making use of the chain rule:

(38) $$\frac{\partial F(\langle r\rangle )}{\partial p}=\frac{\partial F(\langle r\rangle )}{\partial \langle r\rangle }\;\frac{\partial \langle r\rangle }{\partial p}=F^{\prime }\left(\langle r\rangle \right)\;\frac{\partial \langle r\rangle }{\partial p}$$

followed by normalization (19) gives the inferred pdf:

(39) $$p^{*}\left(f\;|\;g,I\right)=\frac{q(f\;|\;g,I)}{Z}\;\exp[-\eta ⊘r\left(f\right)-\zeta ⊘F^{\prime }\left(\langle r\rangle \right)\;r\left(f\right)]$$

where Z=exp(κ+1) is the partition function. Equation (39) is an extended (nonlinear) form of the *Boltzmann distribution*, which can be solved in conjunction with the constraints to give $p^{*}$, *Z*, η and ζ. Once the pdf (39) is available, the *n*th moment of any other uncertain parameter a(f) can also be calculated:

(40) $$\langle a{\left(f\right)}^{n}\rangle =\underset{\Omega_{f}}{⨋}a{\left(f\right)}^{n}\;p^{*}\left(f\;|\;g,I\right)$$

e.g., n=1 gives the mean, while n∈{1,2} gives the mean and variance.

We note that previous treatments of the MaxEnt method, based almost exclusively on linear constraints, give an analytic solution for $p^{*}$, with the Lagrangian multipliers then computed numerically [36,37,38,39,40,41]. In this study, in which (39) contains nonlinear constraints and interdependencies, its solution will usually require an iterative computational scheme. Depending on the problem formulation, nonlinear constraints can give rise to a non-convex Lagrangian, which would preclude the use of the standard tools of convex optimization, e.g., [71], and with the possibility of multiple solutions, e.g., [72]. In the authors’ experience of water distribution, electrical and transport networks, we did not encounter significant computational difficulties or multiple solutions, although we note that the resistance function constraints (6) in such systems tend to be monotonic. We have, however, encountered examples of sharp transitions, for example in pipe flows due to the laminar-turbulent transition, e.g., [32,33], and in transport networks due to routing changes, e.g., [73]. In contrast, for a problem with nonlinear constraints and Gaussian priors, we identified an analytical solution based on matrix operations [74]. We note that a general numerical algorithm for MaxEnt analysis based on nonlinear constraints is not available, and that it may be necessary to implement a tailored solution for a given problem.

If any inequality constraints such as (24) are included in (36), these can be handled by a Lagrangian multiplier which is real-valued at equality and which vanishes when the inequality applies. Usually, an inequality test is implemented at each iteration, enabling the inequality Lagrangian multipliers to be switched on or off as appropriate, e.g., [32]. For some problems, inequality constraints can also be handled using integration limits, e.g., [73]. If any local constraints (21) or (23) are included in (36), they and their corresponding Lagrangian multipliers will be functions of the non-marginalized variables, providing a more elaborate solution than (39), but which accords with the same mathematical framework.

As an example, consider a flow network defined by the standard specifications (10), subject to constraints on normalization (19), global expectation values of some physical properties (20), Kirchhoff’s node laws (29), Kirchhoff’s loop laws (31), resistance functions (32), expected number of species (33) and expected graph properties (35). (Please note that only some expected values (20) should be imposed, otherwise the problem will become overdetermined.) The Lagrangian, for brevity written in expectation form, now becomes:

(41) $$\begin{array}{cc} L & =-\langle \ln\frac{p}{q}\rangle -\kappa \left(\langle 1\rangle -1\right)-\lambda ⊘\left(\langle Q\rangle -v_{Q}\right)-\mu ⊘\left(\langle \Theta \rangle -v_{\Theta }\right)-\nu ⊘\left(\langle ▴E\rangle -v_{▴E}\right)\\ & -\alpha ⊘\left(\langle \hat{\Theta }\rangle +\epsilon \;\Phi \left(\langle \hat{Q}\rangle \right)\right)-\beta ⊘M\left(\langle G\rangle \right)⊘\langle ▴E\rangle -\gamma ⊘\left(\langle ▴E\rangle -R(\langle Q\rangle )\right)-\delta ⊘\Psi \left(\langle C\rangle \right)-\phi ⊘\Xi \left(\langle G\rangle \right) \end{array}$$

where λ,μ,ν,α,β,γ,δ and ϕ are Lagrangian multiplier vectors or matrices of appropriate order and dimension. Combining like terms and taking the functional derivative yields:

(42) $$\begin{array}{cc} p^{*} & =\frac{q}{Z}\;\exp[-\lambda ⊘Q-\mu ⊘\Theta -\nu ⊘▴E-\alpha ⊘\hat{\Theta }-\epsilon \;\alpha ⊘\Phi^{\prime }\left(\langle \hat{Q}\rangle \right)\;\hat{Q}-\beta ⊘M\left(\langle G\rangle \right)⊘▴E\\ & -\beta ⊘M^{\prime }\left(\langle G\rangle \right)\;G⊘\langle ▴E\rangle -\gamma ⊘▴E+\gamma ⊘R^{\prime }\left(\langle Q\rangle \right)\;Q-\delta ⊘\Psi^{\prime }\left(\langle C\rangle \right)\;C-\phi ⊘\Xi^{\prime }\left(\langle G\rangle \right)\;G] \end{array}$$

Depending on the network and its level of description, many of the terms in (42) may simplify further. In principle, (42) can be solved in conjunction with the constraints to give the pdf, the partition function and all Lagrangian multipliers. Since $p^{*}$ is implicit within each expectation, (42) will usually require numerical solution.

### 3.6. Role of Time

We finally comment on the role of time in MaxEnt analysis. In traditional applications in statistical mechanics and in the above formulation, the probabilities, entropy function and constraints are considered independent of time, usually invoking the ergodic hypothesis that the time- and ensemble-averages (25)–(26) are equivalent [57]. However, for many networks it may be desirable to adopt a *time-based* formulation, in which the probabilities, entropy function and constraints are functions of time. Explicitly, instead of (36), the Lagrangian can be written:

(43) $$\begin{array}{cc} L(t) & =-\underset{\Omega_{f}}{⨋}p\left(f\;|\;g,t,I\right)\ln\frac{p(f\;|\;g,t,I)}{q(f\;|\;g,t,I)}-\kappa \left(t\right)\left(\underset{\Omega_{f}}{⨋}p\left(f\;|\;g,t,I\right)-1\right)-\eta \left(t\right)⊘\left(\underset{\Omega_{f}}{⨋}r\left(f\right)\;p\left(f\;|\;g,t,I\right)-v_{r}\left(t\right)\right)\\ & -\zeta (t)⊘F(\langle r(t)\rangle ) \end{array}$$

Philosophically, (43) makes a clear distinction between time and ensemble averaging, essential for the probabilistic representation of a time-dependent dynamical system. The suitability of the MaxEnt algorithm will now depend on the time scales of the network. For simple dynamics, in which the response times of relaxation processes within the network are significantly faster than that of the forcing, the MaxEnt algorithm will provide the “moving stationary state” of the network, or in other words its asymptotic distribution subject to the forcing. If on the other hand, the relaxation processes are significantly slower than the forcing, the network may experience periods of quiescence followed by sudden responses (phase changes or tipping points), for which it may necessary to construct a dynamical model to meaningfully capture the dynamics. For systems with multiple time-scales or more complicated dynamics, the network response to forcing can become very complicated [21]. For such systems, the observer may need to seek insights from both maximum entropy and dynamical models, in the same way that an observer of crystallization or chemical reaction processes will seek insights from both thermodynamics and kinetics.

An alternative *path-based* or *maximum calibre* approach for the analysis of time-varying systems is to redefine the entropy and all constraints as integrals over time [75,76,77], i.e., by integrating (43) with respect to time. The stationary state of the system then becomes the description of its most probable path through time. While this approach is well-defined, with deep connections to the principle of least action [77,78], it requires a very detailed knowledge of the behaviour of the constraints over all times, which is unlikely to be available for most flow networks of interest.

## 4. Applications

The above MaxEnt framework (42) can be used to infer the state of any flow network constrained by mean parameter values, physical laws (such as Kirchhoff’s laws) and/or network properties. This includes the analysis of a wide variety of networks, including pipe flow, electrical, chemical reaction, communications, transportation, epidemiological, human economic and social networks. Since the method is probabilistic, it enables the probabilistic prediction of the flow and other properties of a network when there is insufficient information to obtain a deterministic solution. As noted, the mean properties (or any other moments) of the system can be inferred from the probabilistic solution, by calculating their expected values.

**Figure 2:**
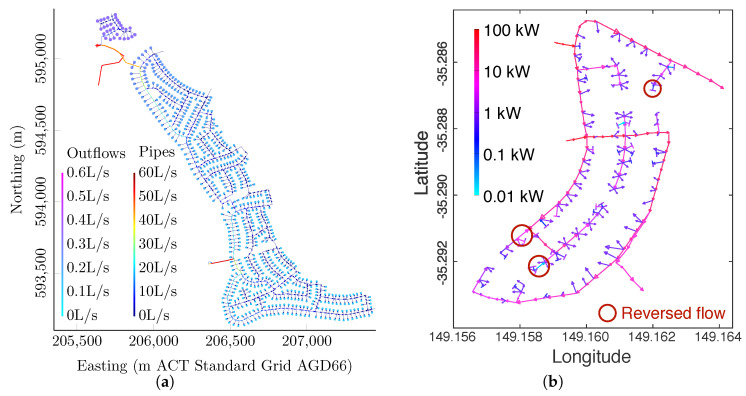
(**a**) Case study water distribution network from Torrens, ACT, Australia, showing inferred flow rates on the network (lines) and delivered to houses (dots), based on constraints on normalization, Kirchhoff’s laws, a grouped inflow rate and a head difference, using a multidimensional Gaussian prior with zero means (after [32] Figure 7a, used with permission), and (**b**) case study electricity grid in Campbell, ACT, Australia, showing inferred mean power flows on the network and to houses, based on constraints on normalization, Kirchhoff’s laws and solar forcing (after [86] Figure 1, used with permission]).

In recent years, variants of the above MaxEnt formulation (42) have been applied to the analysis of a variety of engineered flow networks. These include:

(1) Analyses of pipe flow networks used for water distribution systems, incorporating constraints on normalization (19), linear and nonlinear global expectation values (20) and (22), Kirchhoff’s node and loop laws (29) and (31), and resistance functions (32) [32,33,73,74,79,80,81,82,83,84,85]. This includes development of the analytical formulation, iterative numerical methods to handle nonlinear constraints, and rapid semi-analytical (quadratic programming) schemes for partition function integration. More recently, this work was extended by the use of reduced parameter basis sets to ensure consistency regardless of network representation [33,73,81]. Research also extended to comparative analyses of different prior probability functions [32,33,73,74,79], the use of priors to encode “soft constraints” [73,74,79], and formulation of an alternative linear algebra solution method for nonlinear systems with Gaussian priors [73,74,79]. The method was also demonstrated by application to a 1123-node, 1140-pipe water distribution network from Torrens, ACT, Australia [32,33,73]. An example set of inferred water flow rates on this network is illustrated in Figure 2a; further details of this analysis are given in [32].
(2) Analyses of electrical networks, incorporating constraints on normalization (19), global expectation values (20) and (22), Kirchhoff’s node and loop laws (29) and (31), and resistance or impedance functions (32) [73,86]. This includes analysis of a 400-node electrical network in Campbell, ACT, Australia, subject to solar forcing from distributed household photovoltaic systems. An example set of inferred electrical power flows on this network is illustrated in Figure 2b, showing flow reversals due to high solar forcing; further details of this analysis are given in [73,86].
(3) Analyses of transport networks, reformulated in terms of trip flow rates (from origins to destinations) rather than link flow rates, and incorporating constraints on normalization (19), global expectation values (20) and (22), various forms of Kirchhoff’s node laws (29) and various cost constraints [73,87]. These include different formulations using the gravity model of transport flows, or for route selection by proportional assignment or equilibrium assignment (cost minimization) methods.
(4) Derivation of graph priors for several graph ensembles [88], invoking the relative entropy:
  (44) $$\begin{array}{c} H_{G}=-\sum_{\Omega_{G}}p\left(G|I\right)\ln\frac{p(G|I)}{q(G|I)} \end{array}$$
  where G is reinterpreted to represent a graph macrostate, p(G|I) and q(G|I) are the posterior and prior probabilities and $\Omega_{G}$ is the graph ensemble. This is used in place of the Shannon entropy typically used in MaxEnt analyses of networks, e.g., [6,7,9,10,28,29,30]:
  (45) $$\begin{array}{c} H_{G}^{Sh}=-\sum_{\Omega_{G}}p\left(G|I\right)\ln p\left(G|I\right) \end{array}$$
  in which G represents an individual graph. The use of graph priors enables a simplified accounting over graph macrostates—taking advantage of the most important feature of statistical mechanics—thereby simplifying the analysis of networks with uncertainty in the network structure.

Considerable research is still required on the full extent of the MaxEnt formulation (42) for flow networks, especially for the analysis of multidimensional flows and chemical reaction networks, the formulation of numerical algorithms for particular classes of nonlinear constraints, and the analysis of coupled flow and graphical properties due to uncertainties in both the state and structure of the network.

## 5. Comparison to Bayesian Inference

An alternative method for probabilistic inference is that of Bayesian inference, in which one calculates the probability of an hypothesis or model using Bayes’ rule [64,65]. In the terminology of the present analysis, this gives:

(46) $$p\left(f\;|\;g,I\right)=\frac{p(g\;|\;f,I)p(f\;|\;I)}{p(g\;|\;I)}=\frac{p(g\;|\;f,I)p(f\;|\;I)}{\underset{\Omega_{f}}{⨋}p\left(g\;|\;f,I\right)p\left(f\;|\;I\right)}$$

where p(f|g,I) is the posterior probability or pdf, p(f|I) is the prior probability or pdf of f, p(g|f,I) is the likelihood function, and p(g|I) is the prior probability or pdf of g, commonly termed the evidence. This approach makes use of the Bayesian understanding of a probability as a value between 0 and 1, assigned based on what is known, which need not correspond to a measurable frequency [38]. The application of Bayesian inference to the analysis of flow networks has been studied by many authors, including comparative analyses of the MaxEnt and Bayesian approaches, e.g., [73,74,89,90]. For flow network problems involving moment constraints as formulated here, the MaxEnt method offers the advantage (usually) of simpler mathematical operations compared to the Bayesian method, in which constraints are generally incorporated in a more complicated form, such as delta functions. However, for problems which include nonlinear constraints, this advantage of the MaxEnt method may not be so prominent. For problems involving significant observational data, the Bayesian method will be the more the natural choice, since it allows the incorporation of individual data points rather than summary data expressed in the form of moment constraints.

For Gaussian priors, it can be shown that the MaxEnt and Bayesian methods give the same or very similar posterior mean values, but their covariances are different, e.g., [73,74]. This arises from the different algorithms used: in the Bayesian method, the interactions between variables are applied through the likelihood function, using second or higher-order cross-terms within the posterior pdf. In contrast, the MaxEnt method incorporates interactions between variables using Lagrange multipliers, avoiding second-order correlation terms in the posterior covariance. This suggests that the MaxEnt method could provide a numerical advantage over Bayesian inference for moment-constrained problems of the type examined here, in cases which avoid covariance terms in its integrations.

## 6. Conclusions

We present a generalized MaxEnt framework for inferring the state of a flow network—here defined as a set of nodes and links which carries one or more flows—subject to uncertainty in the parameters of interest and in the network itself. In this method, the network uncertainty is represented by a joint probability function over its unknowns, subject to all that is known. This gives a relative entropy function which is maximized, subject to the constraints, to determine the most probable or most representative state of the network. The constraints can include “observable” constraints on various parameters, “physical” constraints such as conservation laws and frictional properties, and “graphical” constraints arising from uncertainty in the network structure itself. Since the method is probabilistic, it enables the prediction of network properties when there is insufficient information to obtain a deterministic solution. For linear constraints, the MaxEnt framework is analytic, but can also handle nonlinear constraints or nonlinear interdependencies between variables, generally at the cost of requiring numerical solution. The MaxEnt formulation provided can be applied to a large variety of flow networks across many different disciplines, including pipe flow, fluid flow, electrical, chemical reaction, ecological, epidemiological, neurological, communications, transportation, financial, economic and human social networks.
